# Further Evidence Supporting a Role for Gs Signal Transduction in Severe Malaria Pathogenesis

**DOI:** 10.1371/journal.pone.0010017

**Published:** 2010-04-01

**Authors:** Sarah Auburn, Andrew E. Fry, Taane G. Clark, Susana Campino, Mahamadou Diakite, Angela Green, Anna Richardson, Muminatou Jallow, Fatou Sisay-Joof, Margaret Pinder, Malcolm E. Molyneux, Terrie E. Taylor, Kasturi Haldar, Kirk A. Rockett, Dominic P. Kwiatkowski

**Affiliations:** 1 Wellcome Trust Centre for Human Genetics, Oxford, United Kingdom; 2 Wellcome Trust Sanger Institute, Hinxton, United Kingdom; 3 Medical Research Council, Banjul, The Gambia; 4 The Malawi-Liverpool-Wellcome Trust Programme of Clinical Tropical Research, College of Medicine, Blantyre, Malawi; 5 Liverpool School of Tropical Medicine, Pembroke Place, Liverpool, United Kingdom; 6 Blantyre Malaria Project, University of Malawi College of Medicine, Blantyre, Malawi; 7 Department of Internal Medicine, College of Osteopathic Medicine, Michigan State University, East Lansing, Michigan, United States of America; 8 Center for Rare and Neglected Diseases, University of Notre Dame, South Bend, Indiana, United States of America; University of Montreal, Canada

## Abstract

With the functional demonstration of a role in erythrocyte invasion by *Plasmodium falciparum* parasites, implications in the aetiology of common conditions that prevail in individuals of African origin, and a wealth of pharmacological knowledge, the stimulatory G protein (Gs) signal transduction pathway presents an exciting target for anti-malarial drug intervention. Having previously demonstrated a role for the G-alpha-s gene, *GNAS*, in severe malaria disease, we sought to identify other important components of the Gs pathway. Using meta-analysis across case-control and family trio (affected child and parental controls) studies of severe malaria from The Gambia and Malawi, we sought evidence of association in six Gs pathway candidate genes: adenosine receptor 2A (*ADORA2A*) and 2B (*ADORA2B*), beta-adrenergic receptor kinase 1 (*ADRBK1*), adenylyl cyclase 9 (*ADCY9*), G protein beta subunit 3 (*GNB3*), and regulator of G protein signalling 2 (*RGS2*). Our study amassed a total of 2278 cases and 2364 controls. Allele-based models of association were investigated in all genes, and genotype and haplotype-based models were investigated where significant allelic associations were identified. Although no significant associations were observed in the other genes, several were identified in *ADORA2A*. The most significant association was observed at the rs9624472 locus, where the G allele (∼20% frequency) appeared to confer enhanced risk to severe malaria [OR = 1.22 (1.09–1.37); *P* = 0.001]. Further investigation of the *ADORA2A* gene region is required to validate the associations identified here, and to identify and functionally characterize the responsible causal variant(s). Our results provide further evidence supporting a role of the Gs signal transduction pathway in the regulation of severe malaria, and request further exploration of this pathway in future studies.

## Introduction

As an obligatory route to blood parasitaemia, erythrocyte invasion is an essential gateway to malaria disease and a key target for disease intervention. However, we still have only a limited understanding of the molecular mechanisms underlying erythrocyte invasion by *Plasmodium falciparum*, the most virulent species of human malaria parasite, and their impact at the disease level (review in [1]).

A wide spectrum of clinical outcomes is observed in response to malaria infection in endemic regions. While some infected individuals remain asymptomatic, others may suffer mild symptoms of disease, and a small proportion of infections progress to severe, life-threatening complications [2]. As well as environmental and parasite determinants, a number of host factors influence individual response to malarial infection including age/immune status and genetic makeup. These factors may influence the outcome at various stages in the transition from initial infection to blood parasitaemia, fever, severe disease, and death. Thus, factors which promote progression at any of these stages should be enriched in individuals suffering severe disease and death. Case-control association studies using severe disease cases, therefore, present an effective tool to identify host genetic factors regulating any of the earlier transitions in disease progression, including determinants of erythrocyte invasion which regulate blood parasitaemia levels.

Harrison and colleagues demonstrated a role for the host erythrocyte stimulatory G protein (Gs) pathway in erythrocyte invasion [3]. Using peptides designed to disrupt the interaction between the G-alpha-s subunit of the Gs protein and the beta-2-adrenergic receptor, a Gs protein-coupled receptor, reduced erythrocyte invasion by *P. falciparum* parasites and subsequently lower parasitaemia was observed *in vitro* [3]. Seeking evidence of an impact of these Gs signal transduction-related events on disease progression, we previously demonstrated association between polymorphism in the gene encoding G-alpha-s (*GNAS*) and severe malaria disease using a case-control association approach [4]. The demonstration of a disease-regulatory role at the *GNAS* locus warranted further investigation of the Gs pathway to enhance our understanding of the disease mechanism(s) involved and to identify other components which might present suitable targets for anti-malarial drug intervention. Indeed, owing to implications in common disorders such as hypertension and diabetes, the pharmacology of the Gs pathway is well defined [5], meriting it high feasibility for anti-malarial drug intervention. With the availability of the Sequenom iPLEX platform (www.sequenom.com) and the opportunity for lower cost and higher throughput genotyping (∼10-fold increase in SNP number) relative to the previous Sequenom (hME) platform, we were able to undertake economic genotyping in a larger selection of genes, allowing further interrogation of the Gs pathway to identify other disease-regulatory candidates that might present suitable targets for anti-malarial drug intervention.

G protein pathways provide a means for intracellular components to respond appropriately to extracellular stimuli [6]. Different stimuli may require different signal transduction events and subsequent effector responses, and the specificity in the signal transduction systems resides in the G protein coupled receptor (GPCR). Gs proteins transduce signals from several GPCRs to adenylyl cyclases which produce cAMP. Gs GPCRs include adenosine receptor alpha 2A and 2B (ADORA2A and ADORA2B), and beta-1- and beta-2- adrenergic receptors (ADRB1 and ADRB2). Beta-adrenergic receptors are activated by various catecholamines, while adenosine is the preferred agonist for ADORA2A and ADORA2B. Activation of the pathway is initialized by an agonist binding to the appropriate GPCR which has greater affinity for the Gs protein. In the basal state, Gs proteins are heterotrimeric, comprising 3 subunits, alpha, beta and gamma, with GDP bound to the alpha subunit. Stimulation of the Gs protein by interaction with the GPCR results in exchange of GTP for GDP. The GTP-bound G-alpha-s subunit (GNAS) dissociates from the beta-gamma dimer and activates adenylyl cyclase (e.g. ADCY9), which then converts ATP to cAMP. The second messenger, cAMP, activates effector molecules such as protein kinase A, which elicit an appropriate response to the initial agonist stimulation.

Negative feedback processes regulate the Gs pathway in order to prevent excessive cell signalling. Following dissociation from the G-alpha-s subunit, the beta-gamma dimer binds and translocates a G protein coupled receptor kinase such as the beta-adrenergic receptor kinase 1 (ADRBK1) to the membrane [7]. At the membrane, the kinase phosphorylates the agonist-activated GPCR which then complexes with arrestin protein preventing further coupling to the G protein. The free G-alpha-s subunit is deactivated by a regulator of G protein signalling molecule (RGS) such as RGS2, which binds to the G-alpha-s subunit and acts as a GTPase-activating protein to attenuate signalling of the GTP-bound G-alpha-s subunit [8]. RGS2 also appears to interact with ADCY to reduce cAMP production [9].

The inhibitory G (Gi) protein pathway also opposes the effects of the Gs pathway, having an inhibitory effect on adenylyl cyclase and cAMP production. A well studied component of this pathway is the Gi beta subunit 3 (GNB3), which has been implicated in complex disorders such as hypertension [10], [11].

Using meta-analysis pooled across four association studies (Malawi case-control (unrelated cases and controls), Malawi family trio (affected child and parental controls), Gambian case-control and Gambian trio), we tested SNP associations with severe malaria in six genes related to the Gs pathway; adenosine receptor alpha 2A and 2B (*ADORA2A*, *ADORA2B*), beta-adrenergic receptor kinase 1 (*ADRBK1*), regulator of G protein signalling 2 (*RGS2*), adenylyl cyclase 9 (*ADCY9*), and G-protein-beta 3 (*GNB3*). A meta-analysis approach was chosen to enhance sample size whilst allowing for heterogeneity between studies. Owing to the challenges of collecting severe malaria cases in African study sites, large sample sizes are difficult to obtain [12]. Despite efforts to standardize clinical phenotyping across studies, owing to inter-population differences in transmission patterns and demography, some heterogeneity still remains in features such as the proportion of different severe malaria subphenotypes (e.g. severe malarial anaemia).

## Results

### Assay performance

Allele frequencies for the assays (pre-filtered by quality assessment on the HapMap Yoruba dataset) in the Gambian and Malawian studies are presented in Table 1. All assays demonstrated significant concordance with HWE (*P*<0.001) and failure rates below 10% (results not presented).

**Table 1:** SNP allele frequencies in the Gambian and Malawian case-control and family trio studies.

Gene	Assay	1 Alleles	Gambia CC	Gambia Trio	Malawi CC	Malawi Trio
*ADCY9*	rs2238432	C/T	0.28	0.28	0.30	0.28
	rs2230739	A/G	0.16	0.16	0.09	0.08
	rs3730119	C/T	0.26	0.27	0.25	0.25
	rs10775349	C/G	0.20	0.21	0.20	0.21
	rs8047038	C/T	0.31	0.30	0.30	0.30
*ADORA2A*	rs3761422	C/T	0.28	0.28	0.31	0.30
	rs2267076	C/T	0.27	0.27	0.30	0.29
	rs9624472	A/G	0.23	0.23	0.16	0.16
	rs5751876	T/C	0.39	0.40	0.30	0.30
*ADORA2B*	rs2535611	T/C	0.03	0.03	0.13	0.11
	rs11654	G/C	0.32	0.30	0.22	0.23
	rs2286796	G/A	0.37	0.30	0.22	0.23
	rs2302416	G/A	0.29	0.30	0.31	0.33
*ADRBK1*	rs12285582	T/C	0.40	0.40	0.29	0.28
	rs948988	G/A	0.39	0.40	0.28	0.27
	rs7934433	C/T	0.14	0.12	0.28	0.30
*GNB3*	rs3759348	G/A	0.26	0.26	0.29	0.32
	rs5443	T/C	0.18	0.16	0.16	0.16
	rs5446	C/T	0.46	0.49	0.50	0.48
*RGS2*	rs7531013	G/A	0.44	0.42	0.24	0.26
	rs2179652	T/C	0.44	0.38	0.25	0.25
	rs2746073	T/A	0.02	0.01	0.06	0.05

1 Major allele/minor allele. CC: Case-control.

### Single-locus associations

**Figure 1:**
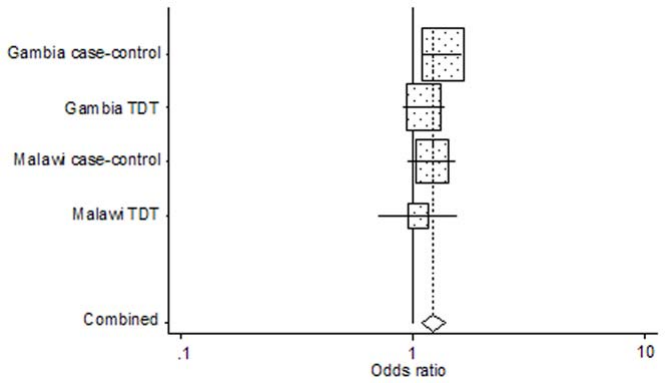
Forest plot of the odds ratios of severe malaria allelic associations at the rs9624472 locus. Odds ratios of severe malaria associations with the rs9624472 G allele are represented for each study as a grey box with a horizontal line. The mid-point of the line represents the odds ratio and the extreme points of the line represent the 95% CI. The contribution of each study to the meta-analysis is represented by the area of the grey box. The solid vertical line presents an odds-ratio of 1 (no association). The diamond at the bottom presents the overall odds ratio and CI: OR = 1.22 (95% CI: 1.09–1.37), P = 0.001. Cochran's Q-test for heterogeneity on 4 degrees of freedom: P = 0.501.

**Figure 2:**
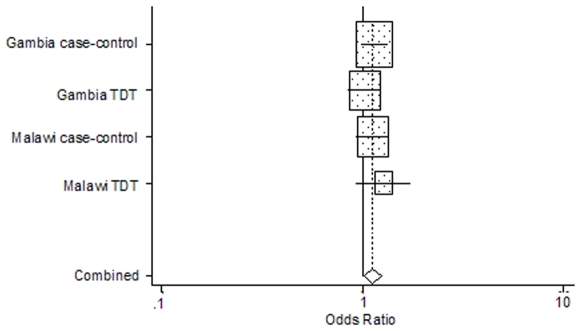
Forest plot of the odds ratios of severe malaria allelic associations at the rs5751876 locus. Odds ratios of severe malaria associations with the rs5751876 C allele are represented for each study as a grey box with a horizontal line. The mid-point of the line represents the odds ratio and the extreme points of the line represent the 95% CI. The contribution of each study to the meta-analysis is represented by the area of the grey box. The solid vertical line presents an odds-ratio of 1 (no association). The diamond at the bottom presents the overall odds ratio and CI: OR = 1.12 (95% CI: 1.02–1.23), P = 0.024. Cochran's Q-test for heterogeneity on 4 degrees of freedom: P = 0.651.

Two of the four *ADORA2A* SNPs, rs9624472 and rs5751876, demonstrated significant association with severe malaria, as illustrated in Figures 1 and 2, respectively. The other two *ADORA2A* loci, rs2267076 and rs3761422, demonstrated a trend of association (0.05≤*P*≤0.1). A summary of the single-locus associations identified in *ADORA2A* are presented in Table 2. The most significant association was demonstrated at the rs9624472 locus (*P* = 0.001), with increased susceptibility to severe malaria conferred by the G allele [OR = 1.22 (1.09–1.37)] in an additive manner [AG OR = 1.17 (1.02–1.36), *P* = 0.029; GG OR = 1.6 (1.13–2.26), *P* = 0.008]. Upon moving up- or down-stream of the rs9624472 locus, the strength of associations weakened. Approximately 2.5 kb downstream of rs9624472, the rs5751876 C allele conferred significant susceptibility to severe malaria [OR = 1.12 (1.02–1.23), *P* = 0.024] with an apparent recessive genotype effect [CT OR = 1.03 (0.89–1.2), *P* = 0.652; CC OR = 1.26 (1.01–1.57), *P* = 0.042]. Four kilobases upstream of rs9624472, the rs2267076 T allele demonstrated a trend of susceptibility to severe malaria [OR = 1.11 (1–1.23), *P* = 0.051] and, further upstream (8 kb), the rs3761422 T demonstrated a weaker trend of susceptibility to severe malaria [OR = 1.09 (0.99–1.21), *P* = 0.096]. No significant associations were demonstrated in the other candidate genes (data in Table S1).

**Table 2:** Summary of pooled single-locus associations observed in the *ADORA2A* gene.

Assay	Coordinate	1Test Variant	OR (95% CI)	Z-value (Pr)
rs3761422	23151226	T (C)	1.09 (0.99–1.21)	1.67 (0.096)
		TT (CC)	1.19 (0.92–1.54)	1.32 (0.187)
		CT (CC)	1.04 (0.90–1.19)	0.51 (0.611)
rs2267076	23155149	T (C)	1.11 (1.00–1.23)	1.95 (0.051)
		TT (CC)	1.30 (0.99–1.71)	1.89 (0.058)
		CT (CC)	1.04 (0.91–1.20)	0.56 (0.573)
rs9624472	23159285	G (A)	1.22 (1.09–1.37)	**3.38 (0.001)**
		GG (AA)	1.60 (1.31–2.27)	**2.65 (0.008)**
		AG (AA)	1.17 (1.02–1.36)	**2.18 (0.029)**
rs5751876	23161855	C (T)	1.12 (1.02–1.23)	**2.26 (0.024)**
		CC (TT)	1.26 (1.01–1.57)	**2.03 (0.042)**
		CT (TT)	1.03 (0.89–1.20)	0.45 (0.652)

Results refer to allele and genotype-based associations pooled across the four studies: Gambia case-control, Gambia trios, Malawi case-control, and Malawi trios.

1 The test variant is followed by the alternative variant in brackets. Odds ratios refer to the effect of the test variant. Significant z-values (*P*<0.05) are presented in bold, trends of association (0.05≤*P*≤0.1) are presented in regular font, and non-significant associations (P>0.1) are presented in dark grey. Cochran's Q-test for heterogeneity on 4 degrees of freedom P>0.05 in all tests.

### Multi-locus associations

Table 3 presents a summary of the meta-analysis associations demonstrated with the common (>5%) *ADORA2A* haplotypes. The two most significant haplotype associations concurred with the single locus associations. The A2A-3 haplotype, which comprises the risk-conferring rs9624472 (G) and rs5751876 (C) alleles, accordingly demonstrated significant risk to severe malaria [OR = 1.21 (1.07–1.37), *P* = 0.002]. Furthermore, the A2A-1 haplotype which comprises the protective alleles at rs9624472 (A) and rs5751876 (T) demonstrated significant protective from severe malaria [OR = 0.86 (0.77–0.95), *P* = 0.003]. With a less significant P-value, the A2A-2 haplotype, which comprises the rs9624472 A and rs5751876 T alleles demonstrated risk to severe malaria [OR = 1.14 (1.02–1.28), *P* = 0.019].

**Table 3:** Pooled haplotype-based odds ratios of association with severe malaria in the *ADORA2A* gene.

Haplotype	Sequence^1^	Study	Freq (%) 2	OR (95% CI)	Z-value (Pr)
A2A-1	1111 (CCAT)	Gambia CC	32.3	0.87 (0.74–1.03)	
		Gambia Trio	30.5	0.94 (0.76–1.17)	
		Malawi CC	40.1	0.84 (0.7–1.01)	
		Malawi Trio	34.2	0.67 (0.48–0.94)	
		**Pooled**		**0.86 (0.77–0.95)**	**−0.30 (0.003)**
A2A-2	2211 (TTAT)	Gambia CC	25.5	1.17 (0.98–1.4)	
		Gambia Trio	24.2	1.19 (0.92–1.53)	
		Malawi CC	28.7	1.08 (0.89–1.31)	
		Malawi Trio	28.5	1.2 (0.82–1.74)	
		**Pooled**		**1.14 (1.02–1.28)**	**2.53 (0.019)**
A2A-3	1122 (CCGC)	Gambia CC	22.4	1.25 (1.03–1.5)	
		Gambia Trio	22.2	1.17 (0.91–1.51)	
		Malawi CC	15.4	1.17 (0.92–1.5)	
		Malawi Trio	18	1.25 (0.78–1.99)	
		**Pooled**		**1.21 (1.07–1.37)**	**3.02 (0.002)**
A2A-4	1112 (CCAC)	Gambia CC	17.5	0.81 (0.66–1.01)	
		Gambia Trio	19.9	0.83 (0.63–1.09)	
		Malawi CC	14.9	0.99 (0.78–1.27)	
		Malawi Trio	18	1.19 (0.74–1.9)	
		**Pooled**		**0.89 (0.78–1.02)**	**−1.67 (0.094)**

Significant z-values (*P*<0.05) are presented in bold.

1 1 = major allele, 2 = minor allele.

2 Frequencies summed across cases and controls. The four haplotypes accounted for over 96% of the variation in each population. Cochran's Q-test for heterogeneity on 4 degrees of freedom P>0.05 in all tests. CC: Case-control.

## Discussion

Owing to implications in the aetiology of erythrocyte invasion by *P. falciparum* [3], [13], evidence of ethnic-specific variants [14], [15], [16], [17] possibly reflecting signatures of selection due to malaria, and a wealth of pharmacological knowledge (review in [5]), the Gs pathway presents an exciting target for anti-malarial drug intervention. Having previously demonstrated a role for the G-alpha-s gene, *GNAS*, in severe malaria disease [4], we sought to identify other important components of the Gs pathway. In this study, and, to the best of our knowledge, the first candidate-based investigation of *ADORA2A* in severe malaria, we demonstrate significant association with this gene using meta-analysis across case-control and TDT studies from The Gambia and Malawi.

We identified a pattern of association in the *ADORA2A* gene whereby single-locus associations strengthened in significance with increasing proximity to rs9624472, at which the most significant association was observed (*P* = 0.001). At this locus, the G allele demonstrated enhanced susceptibility to severe malaria. With respect to the rs9624472 locus, 2.5 kb downstream, the rs5751876 C allele demonstrated susceptibility to severe malaria (*P* = 0.024); 4 kb upstream, the rs2267076 T allele demonstrated a trend of susceptibility to severe malaria (*P* = 0.051); and 8 kb upstream, the rs3761422 T allele demonstrated a weak trend of susceptibility to severe malaria (*P* = 0.096). Using haplotype-based analysis, the two most significant *ADORA2A* haplotype associations concurred with the single-locus associations at rs9624472 and rs5751876; A2A-3 (CCGC) risk to severe malaria (*P* = 0.002), and A2A-1 (CCAT) protection from severe malaria (*P* = 0.003). Association at A2A-2 (TTAT) demonstrated discordance with the single-locus effects of rs9624472 A and rs5751876 T but at lower significance (*P* = 0.019). Further investigation of this region is required to identify the true functional variant responsible for the observed associations.

We used meta-analysis across the Gambian and Malawian TDT and case-control studies in order to enhance our sample size. Across the four studies, we amassed a total of 2278 cases and 2364 controls. A rough estimation of the power of this study to detect the rs9624472 association was 97% at the 5% significance threshold [18]. Nonetheless, with MAF as low as 5%, even this study was not sufficiently powered to detect allelic odds ratios of ∼1.2 (probability of detection ∼44%). With these levels of power, no significant associations were demonstrated with *ADCY9*, *ADRBK1*, *ADORA2B*, *GNB3* or *RGS2*. Further investigation of these genes in other populations and with greater sample size should provide more conclusive evidence on their role in severe malaria pathogenesis.

The results presented here have not been corrected for multiple testing. The application of the Bonferroni correction in this study is debatable as the candidate genes are members of the same biological pathway and, thus, partially interdependent. Due to LD, the SNPs within each gene also exert a measure of interdependence. If we correct the associations observed here by the number of genes tested (6), the threshold for significance is *P*≈*0.008*. At this threshold, the rs9624472 allelic association (*P* = 0.002), and the A2A-1 (*P* = 0.003) and A2A-3 (*P* = 0.002) haplotype associations remain significant.

As a component of a signal pathway, it should be considered that the *ADORA2A* associations presented here may represent a complex network of interactions with other human genetic variants in the Gs pathway such as the *GNAS* locus and, perhaps, other pathways. Indeed, it is feasible that inter-gene interactions may have masked our ability to detect possible associations at the other loci investigated here. However, with regard to multiple hypothesis testing, our study was not sufficiently powered to investigate the numerous possible epistatic interactions between the genes investigated here. In addition to other host genetic factors, it should also be considered that a number of parasite genetic factors and environmental factors may interact with variants in the Gs pathway and, thus, influence the outcome of the disease trait. Indeed, in the context of erythrocyte invasion, which presents an intimate interaction between host and parasite proteins, it is most likely that the composite of numerous parasite and host genetic variants involved in the process ultimately determines the overall rate of invasion. Unfortunately, owing again to limited sample size, this study was underpowered to investigate such variables.

The mechanism by which the *ADORA2A* associations presented here might elicit a pathogenic effect in malaria disease remain unclear. ADORA2A and ADORA2B are both adenosine-activated GPCRs, the latter exhibiting lower affinity for this agonist [19]. The Levels of extracellular adenosine rise in response to metabolic stress and cell damage in conditions such as hypoxia, inflammation and trauma [20], [21]. In addition to erythrocytes, ADORA2A is expressed in a wide range of tissues. Indeed, activation of this receptor in various immune cells appears to promote an anti-inflammatory cytokine profile [22]. A careful balance of pro- and anti-inflammatory cytokine levels appears to be critical to malaria outcome [23]. Thus, in addition to a potential role in regulating erythrocyte invasion, ADORA2A may mediate susceptibility to severe malaria via regulation of inflammatory cytokine levels.

Accumulating evidence, including the *ADORA2A* association reported in this study, indicates that the Gs signal transduction pathway is involved in the regulation of malaria, and more specifically, in the severe, life threatening manifestations of this disease [3], [4], [13]. Thus, this pathway requires further exploration in malaria studies. Indeed, further investigation of the *ADORA2A* gene region is necessary to validate the associations identified here, and to identify and functionally characterize the causal variant(s) responsible for the signals of association. This should enhance our understanding of how ADORA2A is involved in the regulation of severe malaria pathology.

## Materials and Methods

### Ethics Statement

The Gambia Government/Medical Research Council Ethical Committee approved the Gambian samples in this study. The Malawian samples in this study were approved by the College of Medicine Research and Ethics Committee. All samples were obtained with informed written consent from a parent or guardian.

### Samples

Individuals were recruited in a clinical setting. Children under the age of 12 years presenting with severe malaria at the Royal Victoria Hospital in Banjul, The Gambia, and the Queen Elizabeth Central Hospital in Blantyre, Malawi, were enrolled as cases. Severe malaria was defined as severe malarial anaemia (SA), cerebral malaria (CM), other severe complications such as respiratory acidosis, and fatalities due to malaria infection. The threshold for SA was a haemoglobin concentration <5 g/dl in the presence of *P. falciparum* asexual parasitaemia. CM was defined by a Blantyre coma score ≤2, indicating unrousable coma not attributable to convulsions, hypoglycaemia or meningitis in a patient with *P. falciparum* parasitaemia [24]. Due to limited sample size and, thus, power to detect association, severe malaria sub-phenotypes were not investigated here (see Table S2). Population controls for the case-control studies were obtained from umbilical cord blood samples. In the family trios, the patients' mother and father were recruited as controls. Analysis was restricted to true biological trios (≥80% probability of being true parent-offspring trios) confirmed using the *Nuclear* software package [25]. At each study site, local teams managed sample collection and DNA extraction. Genomic DNA samples were subject to whole genome amplification using Multiple Displacement Amplification [26]. DNA from the HapMap Yoruba parental samples (http://www.hapmap.org) was used to test the accuracy of the genotyping assays and was extracted from lymphoblastoid cell lines provided by the Coriell repository (Corriell Institute for Medical Research).

### Gene selection

Genes were selected on account of one or more of the following properties; implication in common disorders which prevail in individuals of African origin possibly as a result of malaria selection [27], such as essential hypertension and type 2 diabetes mellitus [28], [29], [30], [31] prevalence of ethnic-specific polymorphisms which are polymorphic in individuals of African but not Caucasian origin [17]; evidence of functional polymorphism altering signal transduction [32], [33]. We also attempted to investigate the beta-1 and beta-2-adrenergic receptors (*ADRB1* and *ADRB2*), which have previously been implicated in the regulation of *P. falciparum* erythrocyte invasion [3]. However, possibly due to non-unique sequence, we were unable to design accurate genotyping assays on the Sequenom iPLEX and hME platforms for these genes.

### SNP selection

SNP selection was balanced between literature-based evidence of functionality and apparent signatures of selection. This was narrowed down to an economic subset of SNPs for which genotyping assays could be designed on the mass spectrometry iPLEX platform. Three measures were used to identify signatures of positive selection; Wright's Fst [34], haplosimilarity (Hs measure) [35] and extended haplotype homozygosity (EHH measure) [36]. In addition to the genic region, up to 10 kb upstream and downstream of each transcript was investigated (see Table S3). Signatures of selection were sought using the available genotype data from HapMap release no. 19 (http://www.hapmap.org).

### Genotyping methods

Genotyping was undertaken using Sequenom's mass spectrometry iPLEX gold platform (http://www.sequenom.com). Prior to genotyping on the Malawian and Gambian samples, the SNP assays were tested on the Yoruba HapMap parental DNA samples. Only assays with high concordance (>95%) to the official HapMap data (http://www.hapmap.org), genotype distributions conforming to Hardy-Weinberg equilibrium at the 0.1% significance threshold, and high call rates (≥90%) were genotyped on the malaria cases and controls.

### Association analysis

Meta-analysis was undertaken across all the case-control and family studies using the methods outlined in Kazeem and Farrall [37]. Inter-study heterogeneity in association was assessed using Cochran's chi-square test (Q-test) under the null hypothesis of homogeneity (significant heterogeneity *P<0.05*). Individual SNPs were investigated using allele- and genotype-based models. Multi-allele tests were undertaken using haplotypes. Genotype associations were tested using pseudo case-control analysis with correction for HbS using the GenAssoc package (http://www-gene.cimr.cam.ac.uk/clayton/software/). Associations were corrected for the HbS locus, which exerts a strong influence on severe malaria outcome [38], [39]. In the Malawian and Gambian case-control studies, associations were tested using logistic regression analysis and conditional logistic regression with stratification by ethnicity, respectively. Regression analysis was undertaken in STATA (STATA, version 9.0; StataCorp, College Station, TX). In the family trios, association tests were undertaken using the transmission distortion test (TDT) [40].

### Haplotype reconstruction

In the case-control studies, haplotypes were reconstructed using SNPHAP (http://www-gene.cimr.cam.ac.uk/clayton/software/). Only haplotypes with SNPHAP probabilities ≥80% were included in association analysis. In the family trios, haplotypes were reconstructed using an Expectation Maximisation algorithm in R.

## Supporting Information

Table S1 Individual study and pooled odds ratios of allelic associations in all genes investigated. Odds ratios corresponding to severe malaria associations with the minor allele of the given locus are presented for individual studies and pooled studies (meta-analysis). Z scores and corresponding P-values are presented for the pooled associations. Q scores and corresponding P-values are presented for Cochran's test of heterogeneity between the studies. Odds ratios refer to the minor allele at each locus. Cochran's Q-test for heterogeneity on 4 degrees of freedom. CC: Case-control.
(0.30 MB RTF)

Table S2 Severe malaria subphenotype frequencies. CM: Cerebral malaria. SA: Severe anaemia.
(0.04 MB RTF)

Table S3 Properties of Gs pathway candidates. ^1^Region investigated using haplosimilarity (HS) and extended haplotype homozygosity (EHH) tools for detecting signatures of selection.
(0.03 MB RTF)
