# Supplementary material for: Further Evidence Supporting a Role for Gs Signal Transduction in Severe Malaria Pathogenesis
Source: PLoS One. 2010 Apr 1;5(4):e10017. doi: 10.1371/journal.pone.0010017 (PMC2850389; doi:10.1371/journal.pone.0010017)
Supplement: Table S1 — Individual study and pooled odds ratios of allelic associations in all genes investigated. Odds ratios corresponding to severe malaria associations with the minor allele of the given locus are presented for individual studies and pooled studies (meta-analysis). Z scores and corresponding P-values are presented for the pooled associations. Q scores and corresponding P-values are presented for Cochran's test of heterogeneity between the studies. Odds ratios refer to the minor allele at each locus. Cochran's Q-test for heterogeneity on 4 degrees of freedom. CC: Case-control. (0.30 MB RTF) [file pone.0010017.s001.rtf]

Gene	Locus 	Study	Weights (Fixed)	OR (95% CI)	Z score 
(P-value)	1  Heterogeneity Q score (P-value)	
ADCY9 	rs2238432	Gambia CC	122.94	1.22 (1.02-1.45)			
		Gambia Trio	94.96	0.9 (0.73-1.1)			
		Malawi CC	106.78	1.01 (0.84-1.22)			
		Malawi Trio	39.75	0.99 (0.72-1.35)			
		Pooled		1.04 (0.94-1.15)	0.76 (0.45)	5.4 (0.145)	
							
ADCY9 	rs2230739	Gambia CC	83.96	0.95 (0.77-1.18)			
		Gambia Trio	61.38	1.17 (0.91-1.5)			
		Malawi CC	42.23	1.02 (0.76-1.38)			
		Malawi Trio	12.59	0.59 (0.34-1.02)			
		Pooled		1 (0.87-1.15)	-0.03 (0.976)	5.22 (0.156)	
							
ADCY9 	rs3730119	Gambia CC	117.89	1.2 (1-1.44)			
		Gambia Trio	90.6	0.88 (0.71-1.08)			
		Malawi CC	95.38	1.12 (0.92-1.37)			
		Malawi Trio	37.38	1.22 (0.89-1.68)			
		Pooled		1.08 (0.98-1.21)	1.49 (0.137)	5.9 (0.116)	
							
ADCY9 	rs10775349	Gambia CC	100.75	1 (0.82-1.21)			
		Gambia Trio	78.57	0.91 (0.73-1.13)			
		Malawi CC	82.28	0.97 (0.78-1.2)			
		Malawi Trio	32.16	0.9 (0.63-1.27)			
		Pooled		0.96 (0.85-1.07)	-0.79 (0.43)	0.54 (0.909)	
							
ADCY9 	rs8047038	Gambia CC	133.62	1.17 (0.98-1.38)			
		Gambia Trio	90.99	1.02 (0.83-1.26)			
		Malawi CC	106.56	1.07 (0.89-1.3)			
		Malawi Trio	38.99	0.97 (0.71-1.33)			
		Pooled		1.08 (0.98-1.2)	1.51 (0.132)	1.47 (0.688)	
							
ADORA2A	rs3761422	Gambia CC	122.94	1.09 (0.91-1.3)			
		Gambia Trio	91.13	1.07 (0.87-1.32)			
		Malawi CC	108.93	1.07 (0.89-1.29)			
		Malawi Trio	37.68	1.2 (0.87-1.66)			
		Pooled		1.09 (0.99-1.21)	1.67 (0.096)	0.41 (0.938)	
							
ADORA2A	rs2267076	Gambia CC	116.94	1.16 (0.97-1.39)			
		Gambia Trio	89.59	1.09 (0.88-1.34)			
		Malawi CC	106.62	1.05 (0.87-1.27)			
		Malawi Trio	37.26	1.17 (0.85-1.62)			
		Pooled		1.11 (1-1.23)	1.95 (0.051)	0.68 (0.879)	
							
ADORA2A	rs9624472	Gambia CC	105.81	1.36 (1.12-1.64)			
		Gambia Trio	83.98	1.12 (0.9-1.39)			
		Malawi CC	69.63	1.22 (0.96-1.54)			
		Malawi Trio	25.73	1.06 (0.72-1.56)			
		Pooled		1.22 (1.09-1.37)	3.38 (0.001)	2.36 (0.501)	
							
ADORA2A	rs5751876	Gambia CC	145.76	1.15 (0.98-1.35)			
		Gambia Trio	109.74	1.02 (0.85-1.23)			
		Malawi CC	111.86	1.13 (0.94-1.36)			
		Malawi Trio	39.18	1.27 (0.93-1.74)			
		Pooled		1.12 (1.02-1.23)	2.26 (0.024)	1.64 (0.651)	
							
ADORA2B	rs2535611	Gambia CC	19.54	0.72 (0.46-1.12)			
		Gambia Trio	14.41	0.74 (0.44-1.23)			
		Malawi CC	58.8	0.9 (0.7-1.16)			
		Malawi Trio	18.38	1.18 (0.74-1.86)			
		Pooled		0.88 (0.73-1.06)	-1.35 (0.177)	2.87 (0.413)	
							
ADORA2B	rs11654	Gambia CC	133.77	1.1 (0.93-1.3)			
		Gambia Trio	99.61	0.93 (0.76-1.13)			
		Malawi CC	90.45	1.11 (0.9-1.36)			
		Malawi Trio	32.2	0.77 (0.55-1.09)			
		Pooled		1.02 (0.92-1.13)	0.33 (0.744)	4.82 (0.185)	
							
ADORA2B	rs2286796	Gambia CC	125.98	1.01 (0.85-1.2)			
		Gambia Trio	101.98	0.9 (0.74-1.1)			
		Malawi CC	91.87	1.11 (0.91-1.37)			
		Malawi Trio	34.65	0.82 (0.59-1.14)			
		Pooled		0.98 (0.89-1.09)	-0.32 (0.751)	3.47 (0.324)	
							
ADORA2B	rs2302416	Gambia CC	125.14	1.21 (1.01-1.44)			
		Gambia Trio	93.14	0.93 (0.76-1.14)			
		Malawi CC	113.84	1.14 (0.95-1.37)			
		Malawi Trio	41.14	0.75 (0.55-1.02)			
		Pooled		1.05 (0.95-1.17)	1.02 (0.31)	9.07 (0.028)	
							
ADRBK1	rs12285582	Gambia CC	148.58	1.12 (0.96-1.32)			
		Gambia Trio	115.99	0.98 (0.82-1.18)			
		Malawi CC	104.27	0.89 (0.73-1.07)			
		Malawi Trio	44.75	1.01 (0.75-1.36)			
		Pooled		1.01 (0.92-1.11)	0.14 (0.886)	3.55 (0.315)	
							
ADRBK1	rs948988	Gambia CC	148.11	1.12 (0.95-1.32)			
		Gambia Trio	116.48	0.97 (0.81-1.17)			
		Malawi CC	103.59	0.88 (0.73-1.07)			
		Malawi Trio	43.74	0.97 (0.72-1.3)			
		Pooled		1 (0.91-1.1)	-0.05 (0.961)	3.77 (0.288)	
							
ADRBK1	rs7934433	Gambia CC	67.9	0.97 (0.77-1.23)			
		Gambia Trio	49.5	0.82 (0.62-1.08)			
		Malawi CC	95.57	1.14 (0.93-1.39)			
		Malawi Trio	24.13	0.78 (0.52-1.17)			
		Pooled		0.98 (0.86-1.11)	-0.36 (0.718)	4.93 (0.177)	
							
GNB3 	rs3759348	Gambia CC	118.07	1.1 (0.92-1.32)			
		Gambia Trio	92.17	1.06 (0.87-1.3)			
		Malawi CC	109.19	1.15 (0.95-1.38)			
		Malawi Trio	38	1 (0.73-1.37)			
		Pooled		1.09 (0.99-1.21)	1.69 (0.092)	0.63 (0.890)	
							
GNB3 	rs5443	Gambia CC	90.51	1.08 (0.88-1.33)			
		Gambia Trio	59.48	1.03 (0.8-1.33)			
		Malawi CC	67.87	0.89 (0.7-1.13)			
		Malawi Trio	26.13	1.14 (0.78-1.68)			
		Pooled		1.02 (0.9-1.16)	0.29 (0.77)	1.89 (0.597)	
							
GNB3 	rs5446	Gambia CC	156.2	1.03 (0.88-1.2)			
		Gambia Trio	115.16	1.06 (0.88-1.27)			
		Malawi CC	128.89	0.94 (0.79-1.12)			
		Malawi Trio	51.11	1.25 (0.95-1.64)			
		Pooled		1.03 (0.94-1.13)	0.69 (0.488)	3.02 (0.388)	
							
RGS2	rs7531013	Gambia CC	153.18	1.08 (0.92-1.26)			
		Gambia Trio	118.99	0.83 (0.7-1)			
		Malawi CC	95.21	1.01 (0.82-1.23)			
		Malawi Trio	35.25	0.84 (0.61-1.17)			
		Pooled		0.96 (0.87-1.06)	-0.78 (0.433)	5.28 (0.152)	
							
RGS2	rs2179652	Gambia CC	149.89	1.1 (0.94-1.29)			
		Gambia Trio	96.78	0.82 (0.67-1)			
		Malawi CC	97.85	0.95 (0.78-1.16)			
		Malawi Trio	32.81	0.86 (0.61-1.21)			
		Pooled		0.96 (0.87-1.06)	-0.77 (0.441)	5.55 (0.136)	
							
RGS2	rs2746073	Gambia CC	9.08	1.43 (0.75-2.75)			
		Gambia Trio	4.74	1.11 (0.45-2.73)			
		Malawi CC	28.75	1.17 (0.81-1.69)			
		Malawi Trio	8.38	1.27 (0.64-2.49)			
 	 	Pooled	 	1.23 (0.93-1.61)	1.45 (0.146)	0.33 (0.954)	
