# Supplementary material for: Further Evidence Supporting a Role for Gs Signal Transduction in Severe Malaria Pathogenesis
Source: PLoS One. 2010 Apr 1;5(4):e10017. doi: 10.1371/journal.pone.0010017 (PMC2850389; doi:10.1371/journal.pone.0010017)
Supplement: Table S2 — Severe malaria subphenotype frequencies. CM: Cerebral malaria. SA: Severe anaemia. (0.04 MB RTF) [file pone.0010017.s002.rtf]

	Gambia	Gambia	Malawi	Malawi		
Phenotype	Case-control	Family trios	Case-control	Family trios	Total	
CM	550 (62%)	260 (54%)	562 (79%)	142 (72%)	1514	
CM:SA	71 (8%)	137 (28%)	90 (13%)	49 (25%)	347	
SA	212 (24%)	75 (15%)	7 (1%)	2 (1%)	296	
Other Severe	50 (6%)	14 (3%)	53 (7%)	4 (2%)	121	
All Severe	883 (100%)	486 (100%)	712 (100%)	197 (100%)	2278	
Controls	628	972	416	348	2364	
Total	1511	1458	1128	545	4642	
