# Supplementary material for: Further Evidence Supporting a Role for Gs Signal Transduction in Severe Malaria Pathogenesis
Source: PLoS One. 2010 Apr 1;5(4):e10017. doi: 10.1371/journal.pone.0010017 (PMC2850389; doi:10.1371/journal.pone.0010017)
Supplement: Table S3 — Properties of Gs pathway candidates. 1Region investigated using haplosimilarity (HS) and extended haplotype homozygosity (EHH) tools for detecting signatures of selection. (0.03 MB RTF) [file pone.0010017.s003.rtf]

Gene	Genbank (transcript)	Transcript (Kb)	Location	1 Region investigated 	Other genes in region 	
ADORA2A	NM_000675	9.23	22q11.23	23135000-23180000	KIAAO376	
ADORA2B	NM_000676	30.98	17p12	15740000-15860000	TTC19	
ADRBK1	NM_001619	20.27	11q13.2	66740000-66860000	FBXL11, LOC338692, SSH3	
RGS2	NM_002923	3.23	1q31.2	189490000-189535000	-	
GNB3	NM_002075	7.18	12p13.31	6800000-6845000	GRCA2, LEPREL2, CDCA3, USP5, TPI1	
ADCY9	NM_001116	153.53	16p13.3	3925000-4125000	-	
